# The effect of cumulative early life adversities, and their differential mediation through hair cortisol levels, on childhood growth and cognition: Three-year follow-up of a birth cohort in rural India

**DOI:** 10.12688/wellcomeopenres.17712.1

**Published:** 2022-03-03

**Authors:** Debarati Mukherjee, Sunil Bhopal, Supriya Bhavnani, Kamal Kant Sharma, Reetabrata Roy, Gauri Divan, Siddhartha Mandal, Seyi Soremekun, Betty Kirkwood, Vikram Patel

**Affiliations:** 1 Life course Epidemiology, Indian Institute of Public Health-Bengaluru, Public Health Foundation of India, Bengaluru, Karnataka, 560023, India; 2 Population Health Sciences Institute, Newcastle University, Newcastle upon Tyne, NE2 4AX, UK; 3 Maternal & Child Health Intervention Research Group, Department of Population Health, Faculty of Epidemiology & Population Health, London School of Hygiene & Tropical Medicine, London, WC1E 7HT, UK; 4 Child Development Group, Sangath, 451 Bhatkar Waddo, Succor, Bardez, Goa, 403501, India; 5 Center for Chronic Disease Control, C-1/52, 2ND FL, Safdarjung Development Area, New Delhi, 110016, India; 6 Department of Infection Biology, Faculty of Infectious and Tropical Disease, London School of Hygiene and Tropical Medicine, London, WC1E 7HT, UK; 7 Department of Global Health & Social Medicine, Harvard Medical School, 41 Huntington Ave, Boston, MA, 02115, USA; 8 Department of Global Health and Population, Harvard T H Chan School of Public Health, 655 Huntington Ave, Boston, MA, 02115, USA

**Keywords:** adversity, early-life stress, hair cortisol, preschool, DEEP, cognition, growth, LMIC, India

## Abstract

**Background:** Early adversities negatively impact children’s growth and development, putatively mediated by chronic physiological stress resulting from these adverse experiences. We aimed to estimate the associations between prospectively measured cumulative early adversities with growth and cognition outcomes in rural Indian preschool children and to explore if hair cortisol concentration (HCC), a measure of chronic physiological stress, mediated the above association.

**Methods:** Participants were recruited from the SPRING cRCT in rural Haryana, India. Adversities experienced through pregnancy and the first year of life were measured in 1304 children at 12-months. HCC was measured at 12-months in 845 of them. Outcome measures were height-for-age-z-score (HAZ), weight-for-age-z-score (WAZ) and cognition, measured in 1124 children followed up at 3-years. Cognition was measured using a validated tablet-based gamified tool named DEEP.

**Results:** Cumulative adversities at 12-months were inversely associated with all outcomes measures at 3-years. Each unit increase in adversity score led to a decrease of 0·08 units [95% confidence interval (CI):-0·11,-0·06] in DEEP-z-score; 0·12 units [-0·14,-0·09] in HAZ and 0·11 units [-0·13,-0·09] in WAZ. 12-month HCC was inversely associated with DEEP-z-score (-0·09 [-0·16,-0·01]) and HAZ (-0·12 [-0·20,-0·04]), but the association with WAZ was not significant (p = 0·142). HCC marginally mediated the association between cumulative adversities and HAZ (proportion mediated = 0·06, p = 0·014). No evidence of mediation was found for the cognition outcome.

**Conclusions:** Cumulative early adversities and HCC measured at 12-months have persistent negative effects on child growth and cognition at 3-years. The association between adversities and these two child outcomes were differentially mediated by HCC, with no evidence of mediation observed for the cognitive outcome. Future studies should focus on other stress biomarkers, and alternate pathways such as the immune, inflammation and cellular ageing pathways, to unpack key mechanisms underlying the established relationship between early adversities and poor child outcomes.

## Introduction

At least 250 million children in low- and middle-income countries (LMICs) are failing to reach their developmental potential due to multiple adversities experienced during early life ^1^. Early adversities could have life-long consequences such as mental ill-health ^2, 3^, poor school readiness and academic achievement ^4^, and possible adoption of maladaptive behaviors ^5^, which not only compromise growth, development and health through childhood, adolescence and adulthood, but also impact nurturing care of the next generation, leading to continuing cycles of disadvantage.

With the growing recognition that adversities typically co-occur ^6^, recent literature has begun to focus on the impact of cumulative adversities. The seminal adverse childhood experiences (ACEs) studies which retrospectively assessed multiple early adversities found positive associations between cumulative adversities experienced during childhood with a range of adverse adult health outcomes ^7– 10^, many of which are the biggest contributors to the global burden of diseases ^11^. In children, cumulative adversities are predictive of brain functioning ^12^, general cognitive ability ^13– 15^, executive functioning ^14, 16^, social-emotional outcomes ^17, 18^, and several psychopathologies in adolescence ^19, 20^. This epidemiological evidence is supported by neuroscientific findings linking early adversities with structural and functional impairments in developing brain networks ^21– 23^.

This study aimed to address two key knowledge gaps. First, while cumulative adversities are likely to adversely affect both physical growth (height and weight) and cognitive outcomes in children, prospective studies that allow comparing the effects of cumulative early adversities on later growth and cognition in the same sample are missing. The differential effects of different types of adversities on these two outcomes are also largely unexplored.

Further, one of the mechanisms through which early adversities are postulated to lead to poor child outcomes is through the dysregulation of the hypothalamus-pituitary-adrenal (HPA) axis which controls an individual’s response to stressful situations. While earlier studies have examined salivary cortisol as a marker of physiological stress ^24^, hair cortisol concentration have recently gained prominence as a reliable marker of chronic HPA axis activity in children ^25– 29^. However, the prospective association between hair cortisol levels with later child outcomes has been described in only three studies ^30– 32^, with common childhood diseases, child mental health, and physical and mental well-being being the primary outcome measures. Second, the putative causal mediation role of hair cortisol levels on the association between cumulative adversities and later growth and cognitive outcomes in children has not been investigated so far.

This study aims to contribute to bridging these knowledge gaps by addressing the following primary objectives: (1) estimate and compare the associations of cumulative early adversities measured through pregnancy and the first year of life with physical growth (height-for-age-z score and weight-for-age-z-score) and cognition measured two years later when children were three years old; (2) estimate the association between hair cortisol levels at one year with growth and cognition at three years; and (3) as a secondary objective, explore the potential mediating role of hair cortisol levels on the associations between early adversities and later growth and cognitive outcomes, if significant associations were found for (1) and (2). We hypothesize that cumulative early adversities will be inversely associated with growth and cognitive outcomes and these associations will be mediated by chronic stress as measured by hair cortisol levels.

## Methods

### Patient and public involvement statement

Patients or the public were not involved in the design, conduct, reporting, or dissemination plans of this study.

### Study population

Participants were from the SPRING cluster randomized controlled trial which recruited children from 120 villages in Rewari district of rural Haryana, India. The SPRING program has been described in detail elsewhere ^33, 34^. Participating families were identified by a trial surveillance system whereby trained fieldworkers visited every household in the study area every 8 weeks to identify and enrol pregnancies and births, and to follow up those enrolled through the first two years of life. In total, 7015 families were enrolled by the surveillance system from 24 clusters, defined as the catchment area of a functional primary health sub-centre which typically caters to about 6000 individuals. Overall, 5114 children born on or after 18 ^th^ June 2015 were included in the trial since the SPRING intervention had been fully implemented by then. Outcome measures (height-for-age z-score (HAZ) and neurodevelopment measured using the Bayley’s Scales of Infant and Toddler Development, 3 ^rd^ Edition (BSID-III)) were assessed in 1443 children at 18 months of age.

### Data collection

SPRING-ELS (early life stress) was a nested sub-study of the SPRING trial evaluating the effects of early adversities and stress on child growth and development at 12 months of age ^35, 36^. As part of the sub-study, adversities experienced during pregnancy and the first year of life were assessed at 12 months in 1304 children. All assessments were done in the household within -7 to +21 days of the child’s first birthday, except for socioeconomic status which was assessed during enrolment into the study. Adversity assessments were done using questionnaires and observation of the mother and child by trained assessors. Hair samples were collected to measure cortisol concentration from all consenting children with adequate amounts of hair at 12 months of age.

In total, 1259 of the 1443 children who had completed the SPRING outcome assessment at 18 months were followed-up when they turned 3-years old between August 2018 – March 2019. Children’s height, weight and cognitive development was measured by eight trained assessors in children’s homes in the presence of their primary caregivers.

### Details of measures

Exposure variables (measured through pregnancy and the first year of life)

*Cumulative adversity:* A set of 22 contextually relevant adversities were selected based on interviews with the families, discussion with child development experts and review of relevant literature (see Table 1 for a complete list of selected adversities). Since children at 12 months typically spend most of their time in the home and interact with close family members, adversities operating at the level of the household were prioritized. The 22 adversity measures were categorized into four domains based on their proximity to the child, and presented as a conceptual model described earlier ^36^. Factors most distal to the child included household level **Socio-economic factors (SES)** that are known to indirectly affect child growth and development. The next set of factors operated at the level of the primary caregiver were operationalized as **Maternal stress**. Quality of **Relationships** of the child with their caregivers was the third domain and finally, the most proximal domain were factors that were direct stressors to the **Child**. Six factors each were assessed within the SES, Maternal Stress and Child domains, while the Relationship domain comprised four factors as listed in Table 1. 

**Table 1.** Prevalence of adversities categorized across four domains and the proportion of missing values imputed. a Socio-economic status (SES) score calculated with principle components analysis using household demographics and animal & asset ownership b Answered yes to question: “Since you became pregnant, have you or your immediate family who live with you been in debt?” c Answered yes to question: “Since you became pregnant, have you ever been hungry because you could not afford to buy food?” or similar related to child d Using World Health Organization (WHO) multi-country study on women’s health and domestic violence against women e If woman reported husband drinking alcohol, answered yes to question: “does this cause any problems for you” f Question: “When [person] found out your baby was a girl were you/they happy, unhappy or didn’t mind whether you had a girl or a boy?” g Assessed using observed feeding index. Very low quality means *<* = 1 positive verbalisations, and *<* = 1 games played and *<* = 1 responsive actions, plus *>* = 1 negative actions by mother towards child during feeding session h The Home Observation for the Measurement of the Environment Inventory i Not exactly 20% because cut-off made at HOME score of 27 *E All items were assessed at 12 months of age except those marked *E which were collected at enrolment into the surveillance system

Domain	Items	Prevalence (%)	Imputation
Socioeconomic	1. Socioeconomic status: lowest quintile of asset index (*E) ^a^ 2. Father education level: primary or none (*E) 3. Mother education: none or 1-5 grades (*E) 4. Father occupation: at home, seasonably employed or casual labourer 5. Mother married under legal age (18 years) 6. Family debt ^b^ or mother reports being unable to afford food for self or child at any point ^c^	21·5 5·3 12·3 24·5 19·1 17·7	26 (2·3%)
Maternal Stress	1. Mother reports death of husband, parent, sibling, child or friend since pregnancy 2. Mother seriously injured or ill since pregnancy 3. Any violence from husband or mistreated by any other person since pregnancy ^d^ 4. PHQ9 score >=5 or problems described make it very/extremely difficult to do daily activities 5. Duke social support & stress scale: support <=40 or stress >27 6. Husband’s alcohol use causes problems for mother ^e^	5·3 4·4 13·4 19·2 6·9 8·5	19 (1·7%) 19 (1·7%)
Relationship	1. Any of mother, father, mother or mother-in-law were “unhappy” when found out child was a girl ^f^ 2. Mother’s Object Relations Scale concern level: moderate or high 3. Observed feeding style: very low quality ^g^ 4. HOME inventory ^h^ score: lowest quintile ^i^	15·0 50·5 8·1 15·8	371 (33·0%) 1 (0·09%)
Direct child stressors	1. Mother-reported child born prematurely 2. Child admitted to hospital any time during first year of life 3. Mother & child separated for one week or more during first year of life 4. Child left alone or with child under 10 years for more than one hour in the past week 5. Older children who live in house: say anything to make child cry or unhappy (in last week) 6. Older children who live in house: hit/punched/kicked/bit child on purpose to make them unhappy (in last week)	10·3 14·9 1·8 4·5 31·7 18·4	

Adversity scores were derived in three different ways to assess total and domain specific effects of cumulative adversities on outcomes as described below.

*Cumulative adversity score:* Sum of all adversities experienced by the child, treated as a continuous variable. This represented the cumulative adversity total score with a possible range of 0–22. Since less than 5% of the sample experienced 8 or more adversities simultaneously, they were grouped together as the 8+ category in regression analyses.

*Domain specific adversity scores:* Sum of all adversities experienced within each domain (SES, Maternal stress, Relationships and Child), treated as a continuous variable. This represented the cumulative adversity domain specific score and was used to examine the relative influence of the different domains of adversities on child outcomes. Possible score ranges were 0–6 for SES, Maternal stress, and Child domains, and 0–4 for Relationships.

*Adversity (PCA score) quintiles:* Since adversities are known to cluster within households ^6^, a principle component analysis (PCA) approach was used to determine the linear combination of factors that contributed to the maximum variance in the data, to avoid misinterpretation due to underlying collinearity between adversity measures. The raw PCA score of the first principle component (PC1) categorized into quintiles formed the third explanatory variable. 

Chronic stress: The detailed protocol for hair sampling and hair cortisol measurements has been previously described ^35^. Briefly, hair samples were collected at 12 months from the posterior vertex, which is the area with the least intra-individual variability ^37^. Assessors aimed to collect at least 10 mg of hair, which translated to 2–3 cm length of hair from a 1-cm diameter area on the head. Once collected, the samples were wrapped in aluminium foil with the scalp end marked. Subsequently, 3-cm of hair most proximal to the scalp was cut, repackaged into aluminium foils and paper envelopes, and shipped to the laboratory at room temperature for hair cortisol measurement. In the laboratory, cortisol extraction and analysis involved washing the hair in isopropanol, drying them for 24 hours, cutting them fine with standard scissors, and analysis using a Salimetrics ELISA kit (#1-3002) as per the manufacturer’s instructions (Salimetrics USA). Results were converted into picograms of cortisol per mg of hair. Log-transformed hair cortisol concentrations (since the distribution was left-skewed), treated as a continuous variable, was the fourth explanatory variable.

Outcome variables (measured during the 3-year follow-up assessment)

*Anthropometry:* Height was measured using a Seca 213 Portable Stadiometer and weight by SECA-384 electronic scale following World Health Organization (WHO) protocols ^38^. The raw height and weight data were age- and gender adjusted using WHO norms to derive height-for-age (HAZ) and weight-for-age (WAZ) z-scores ^38^. They comprised the first set of outcome variables representing physical growth.

**Figure 1.**
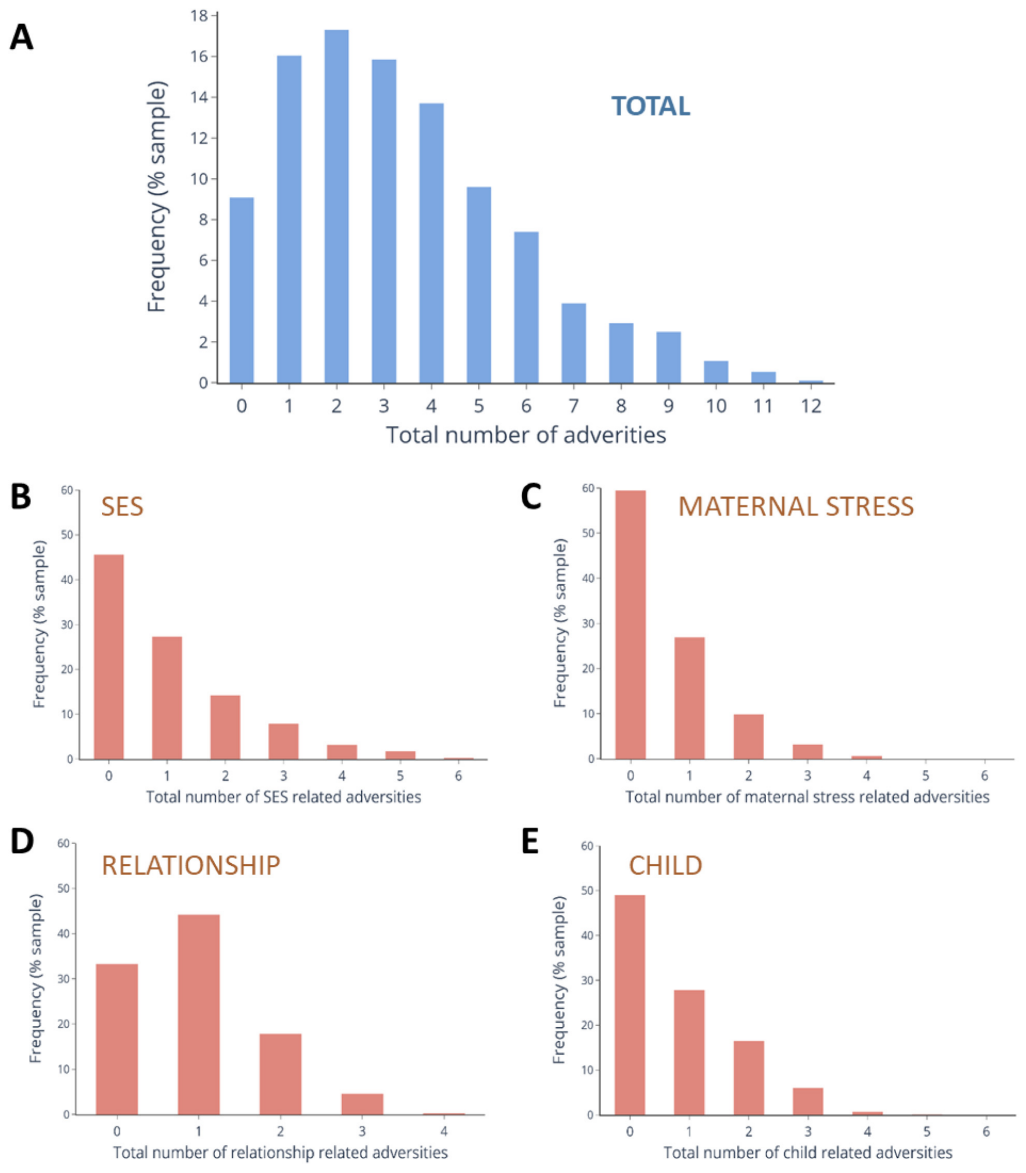
Distribution of total and domain specific adversity scores in the sample. Distribution of total adversity scores in the sample (N = 1124) across ( **A**) all domains and for each sub-type of adversity. They are ( **B**) Socio-economic status (SES) ( **C**) Maternal stress, ( **D**) Relationships, and ( **E**) Child factors.

*Cognition:* Cognition was measured using a novel tablet-based tool named Developmental Assessment on an E-Platform (DEEP). DEEP is a gamified assessment of cognitive abilities administered on a low-cost Android device (Samsung Tab E) by non-specialists in the comfort of the child’s home ^39^. In the version used in this study, DEEP comprised nine games that assessed multiple domains of cognition such as processing speed, manual coordination, response inhibition, divided attention, reasoning, visual integration and visual form perception. The list of games on DEEP, example screenshots, and the main instructions for gameplay are demonstrated in Extended Data - Fig. 1 ^40^. DEEP metrics have been demonstrated to be predictive of a child’s BSID-III cognitive score using a supervised machine learning analysis approach ^41^. DEEP scores in this sample show significant positive associations with concurrently and prospectively measured HAZ ^42^, which is one of the most commonly used proxy indicators of cognition ^43, 44^. DEEP scores were scaled to z-scores to allow comparison of the effects of cumulative adversities on physical growth versus cognition.

Since assessment on DEEP required children to interact with a tablet computer and understand the rules of the game as verbally described by the assessor, children with severe visual or hearing impairments, or any other condition that affected their meaningful interaction with a tablet computer were excluded from the three-year assessment. Approximately 10% of all visits by trained assessors were supervised by a field supervisor. Weekly group sessions were conducted with all assessors to provide feedback. Regular refresher trainings were provided by senior team members.

### Statistical analysis

Data were analysed in STATA version 15 (StataCorp LLC, College Station, TX, USA, RRID:SCR_012763) and the R statistical software (RRID:SCR_001905) ^45^. Missing values in the explanatory variables were imputed using Multiple Imputation by Chained Equations (MICE) separately for the analyses relating to the three outcome variables (DEEP-z-score, HAZ, WAZ).

*Associations between exposure and outcome variables:* The associations between the adversity and hair cortisol exposure variables measured at 12 months (continuous variables) with growth and cognition outcomes at 3 years (continuous variables) were determined using multilevel modelling, with cluster as the random effect and intervention allocation arm included as a fixed effect. Age at 3-year assessment and gender were included as potential confounders. The predicted means and 95% confidence intervals (CI) of the outcome measures (HAZ, WAZ and DEEP z-scores) for each level of adversity (total cumulative, domain specific cumulative and quintile) or log-cortisol measures (mean and 2SDs above and below the mean) were calculated from the adjusted regression models and plotted to visualize the change in growth and cognition outcomes with increasing levels of adversity and stress. For domain specific cumulative adversities, results were plotted as the predicted mean outcome z-score for zero adversities within the domain, along with the slope indicating the unit change in outcome z-score per unit increase in domain specific adversity.

*Exploratory mediation analysis:* We hypothesized that stress resulting from experience of early adversities, using the proxy biomarker of hair cortisol concentration, mediates the association between cumulative adversities (total score) and child outcomes at 3-years. Causal mediation analysis was conducted using the ‘mediation’ package in R ^46^. Multilevel models, with cluster as the random effect, intervention allocation arm as the fixed effect and age at 3-year assessment and gender as confounders were generated between [1] exposure and outcome, [2] exposure and mediator, and [3] exposure and outcome adjusted for the mediator. Effect estimates were compared between models [1] and [3]. Causal mediation was determined using the ‘mediate’ function within the mediate package on models [2] and [3]. Results were tabulated to report the average causal mediated effect (ACME), the average direct (ADE), the total effect, and the proportion of effect that is mediated, along with their respective p-values.

### Ethics

This study was conducted in accordance with the Declaration of Helsinki. Written informed consent was taken from parents during enrolment in the SPRING study, and before the 18- and 36-month outcome assessments. Ethics approval for the SPRING/ELS study for assessments conducted at 12 months was obtained from the London School of Hygiene & Tropical Medicine research ethics committee (23 June 2011; approval number 5983) and the Sangath Institutional Review board (IRB) (19 February 2014). Approval was also granted by the Indian Council of Medical Research’s Health Ministry Screening Committee (HMSC) (24 November 2014). Ethical approval for data collected for the follow-up study at 3-years was obtained from the Public Health Foundation of India (PHFI) (27 October 2017; 18 July 2018) and Sangath IRBs (23 August 2018). LSHTM provided ethics approval for secondary analysis of the SPRING dataset for this follow-up study (11 June 2020; approval number 9886 (5983) – 6).

### Role of the funding source

The funding agencies had no involvement in the collection, analysis, and interpretation of data, writing of the report and the decision to submit the paper for publication. 

## Results

### Description of the study sample

Of 1304 children with adversity assessments from the SPRING-ELS study, we assessed 1124 (86.2%) for outcomes at 3-years. Loss-to-follow-up was due to: moved away (92); temporarily unavailable (37); not approached because lost-to-follow-up at 18-month SPRING outcome assessment (32); withdrew or refused consent (11); technical errors with DEEP data (5); inability to engage with DEEP assessment (2); and death of child (1). Participant demographics of the sample that was lost to follow-up was largely comparable with the sample that was followed up, except for a slightly lower proportion of boys and higher proportion of parents with graduate level education ( Table 2). Child sociodemographic characteristics during the 3-year follow-up assessment show that mean age was 38·8 months (SD: 0·97; range = 34–42 months). 45·6% were female. 44·7% attended preschool. 71·2% of mothers and 67·8% of fathers had completed at least secondary or higher secondary schooling, and the sample was equally distributed across the SES quintiles computed at the time of enrolling families into the SPRING study during 2014–15.

**Table 2.** Participant demographic details. *LTFU = Loss to follow-up, SES = Socio-economic status.

Characteristic	N = 1124 (adversity and DEEP)	N=607 (cortisol and DEEP)	N = 180 (LTFU * at 3-years)
Male, n (%)	611 (54·4)	294 (48·4)	83 (46·1)
Age at 3-year assessment (months), mean (SD)	38·8 (0·97)	38·8 (0·97)	-
Mother’s age at delivery (years), mean (SD)	22·4 (3·8)	22·4 (3·7)	22·2 (3·6)
Mother’s education level, n (%) Below primary (including never been to school) Primary/middle school completed Secondary/higher secondary school completed College & above	70 (6·2) 366 (32·6) 434 (38·6) 254 (22·6)	32 (5·3) 183 (30·2) 240 (39·5) 152 (25·0)	11 (6·1) 40 (22·2) 72 (40·0) 57 (31·7)
Father’s education level, n (%) Below primary (including never been to school) Primary/middle school completed Secondary/higher secondary school completed College & above	25 (2·2) 258 (23·0) 503 (44·8) 338 (30·1)	11 (1·8) 135 (22·2) 271 (44·7) 190 (31·3)	2 (1·1) 28 (15·6) 82 (45·6) 68 (37·8)
SES quintile (during enrolment), n (%) Q1 (poorest) Q2 Q3 Q4 Q5 (wealthiest)	242 (21·5) 251 (22·3) 230 (20·5) 220 (19·6) 181 (16·1)	126 (20·8) 120 (19·8) 144 (23·7) 120 (19·8) 97 (16·0)	38 (21·1) 28 (15·6) 32 (17·8) 35 (19·4) 47 (26·1)
Height-for-age (z-score), mean (95% CI) at 3-year assessment Stunted, n (%)	-1·59 (0·99) 35·6	-1·54 (1·01)	-
Weight-for-age (z-score), mean (95% CI) at 3-year assessment Underweight, n (%)	-1·41 (0·94) 23·3	-1·35 (0·96)	-
Preschool enrolment, n (%) Private preschool Anganwadi centers Other None	266 (23·7) 219 (19·5) 17 (1·5) 622 (55·3)	165 (27·2) 115 (19·0) 9 (1·5) 318 (52·4)	-

At three years, mean (SD) HAZ and WAZ were -1·59 (0·99) and -1·41 (0·94), respectively. Overall, 35·6% were stunted, and 23·3% underweight (19·3% were both) as per WHO definition ^38^. Mean (SD) cognitive score (DEEP) was 69·8 points (SD: 3·09; range: 60·5 – 79·2). 2·6% of the sample fell outside two standard deviations from the mean.

Of the 1304 children with adversity data at 12-months, hair cortisol data was available for 845 children, and of those, 607 were followed-up at 3-years. The geometric mean (SD) of hair cortisol concentration was 64·56 (10·71) picograms/mg of hair (Range: 0·44 – 103,934 pg/mg). Participant demographics of the sub-sample of participants with hair cortisol data (N=607) were similar to those of the larger sub-sample of 1124 children ( Table 2).

### Prevalence of cumulative adversities

The prevalence of each individual adversity is listed in Table 1. In total, 102 (9·1%) children did not experience any adversity, whereas 57·6% experienced 3 or more ( Figure 1A). Mean total adversity score was 3·36 (range 0–12) ( Figure 1A). The maximum possible score within individual domains of adversity was 6, apart from Relationship which was 4. The range of the scores in our sample were: SES: 0–6; Maternal stress: 0–4; Relationship: 0–3; Child: 0–5 ( Figure 1 B–E).

### Prospective association between cumulative adversities and cognition

**Figure 2.**
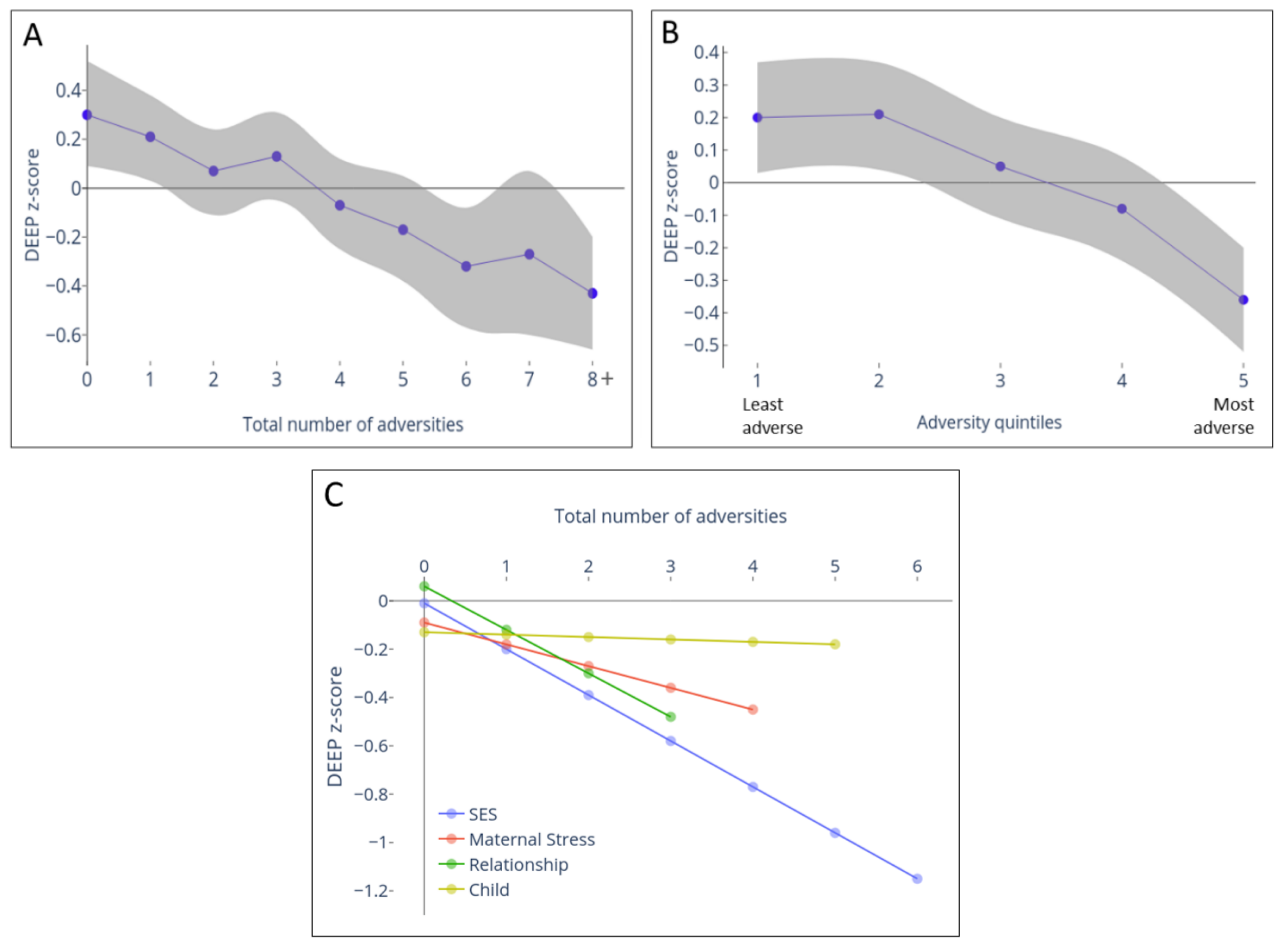
Relationship between cumulative adversity measured at 12 months and cognition measured at 3 years using Developmental Assessment on an E-Platform (DEEP). Cognitive development was measured using a gamified tablet-based tool named DEEP. DEEP scores were transformed to z-scores to interpret the impact of increasing levels of adversity on SD units change in DEEP scores. The predicted mean (95% confidence interval [CI]) DEEP z-score was computed from mixed-effects linear regression models adjusted for gender and age at 36-month assessment. ( **A**) Relationship between the mean predicted DEEP z-score (purple) with 95% CI (grey shaded area) when adversity was measured as the summed score or ( **B**) categorized into quintiles based on principle component analysis (PCA) score is represented. ( **C**) Mean predicted DEEP z-score for zero adversities experienced within each domain (SES, Maternal stress, Relationships, Child), and the linear decline per unit increase in adversity within each sub-type of adversity. The total number of adversities measured within each domain was 6, except for Relationship which measured 4.

There was a significant inverse association between cumulative adversity total score at 12 months and cognition at 3-years ( Figure 2 and Extended Data-Table 1 ^40^). This was approximately linear with no threshold or ceiling effects ( Figure 2A); each unit increase in adversity being associated with a decrease of 0·08 units in DEEP z-score (95% CI: -0·11, -0·06; p-trend < 0·001). The predicted mean DEEP z-scores of children experiencing none versus eight or more adversities were +0·30 and -0·43 respectively, resulting in a difference of 0·7 SDs between these groups.

A similar relationship was observed when adversity scores were categorized into quintiles based on PCA scores ( Figure 2B). The difference in mean predicted DEEP z-scores between the extreme quintiles was 0·56 SDs (Extended Data-Table 1 ^40^). A threshold effect was observed for the two lowest adversity quintiles; beyond this, a sharp linear decrease in DEEP z-scores was observed for each additional adversity quintile ( Figure 2B, p-trend < 0·001). The sharpest drop in predicted DEEP z-score was between groups experiencing the highest levels of adversity (i.e. moving from the fourth to fifth quintile resulted in a decrease of 0·28 units in DEEP z-score, compared to no difference between the first and second quintiles; Extended Data-Table 1 ^40^).

### Prospective association between cumulative adversities and physical growth

**Figure 3.**
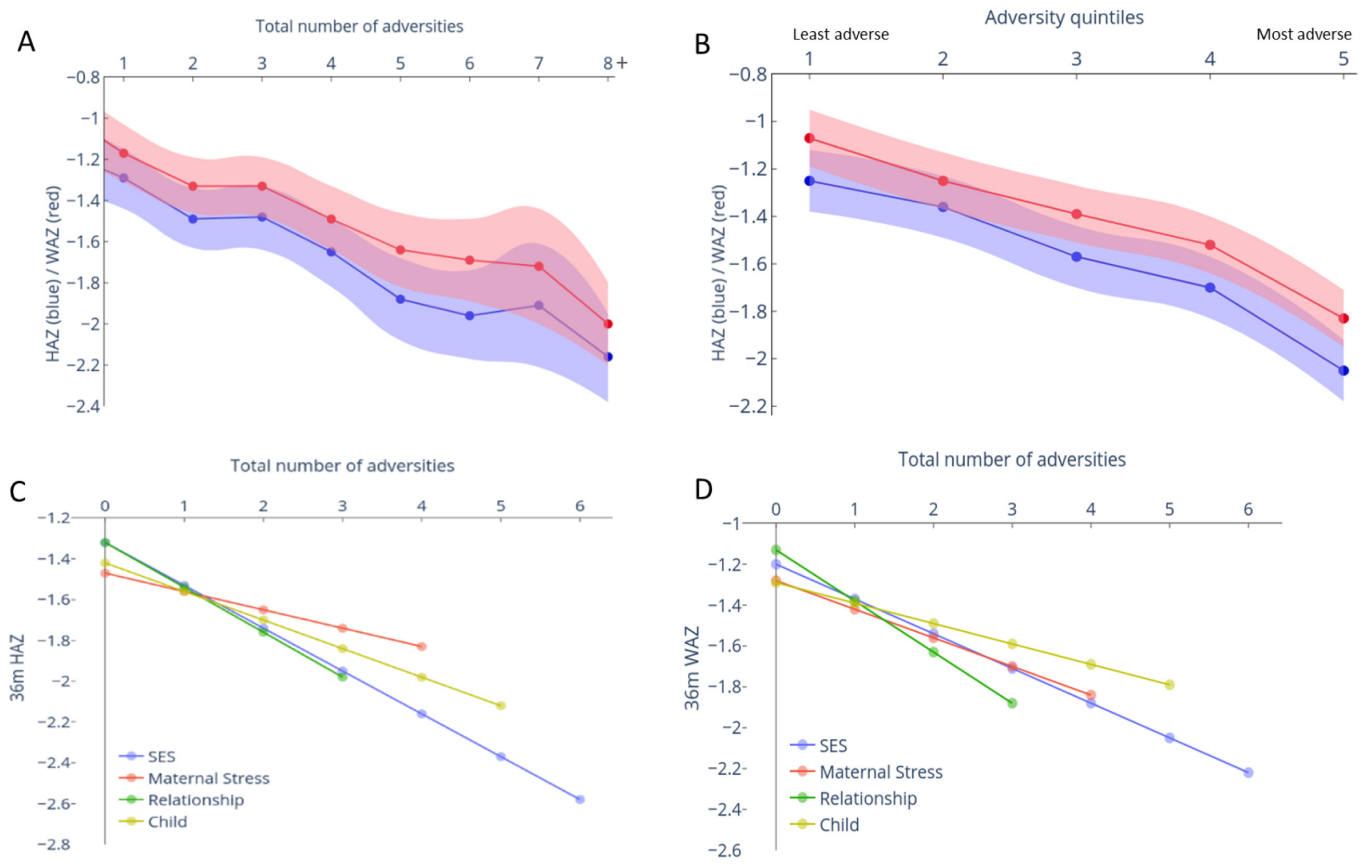
Relationship between adversity measured at 12 months and anthropometric measures at 3 years. The predicted mean (95% confidence interval [CI]) height-for-age z-score (HAZ) and weight-for-age z-score (WAZ) was computed from mixed-effects linear regression models adjusted for gender and age at 36-month assessment. ( **A**) Relationship between the mean predicted HAZ (blue) and WAZ (red) with 95% CI (shaded area) when adversity was measured as the summed score or ( **B**) categorized into quintiles based on principle component analysis (PCA) score is represented. The mean predicted HAZ ( **C**) or WAZ ( **D**) for zero adversities experienced within each sub-type of adversity is plotted, along with the linear decline per unit increase in adversity within each domain.

The association between early adversities, represented both as cumulative total scores or quintiles, and anthropometric measures (HAZ, WAZ) at 3-years showed similar inverse relationships as seen with children’s cognitive outcomes ( Figure 3A, B). Specifically, each unit increase in cumulative adversity total score led to a decrease of 0·12 (95% CI: -0·14, -0·09; p < 0·001) units in HAZ and 0·11 (95% CI: -0·13, -0·09, p < 0·001) units WAZ at 3-years ( Figure 3 and Extended Data-Table 1 ^40^). The difference in the predicted mean HAZ and WAZ scores between groups of children experiencing none versus 8+ adversities was 1·05 SDs and 1·01 SDs, respectively, and the difference between the least and most adverse quintiles were 0·76 and 0·8 SDs respectively (Extended Data-Table 1 ^40^). Unlike the threshold effect seen with cognitive outcomes for the first two adversity quintile groups, a linear decrease was observed across all groups for HAZ and WAZ outcomes.

### Relative effect of different adversity subtypes on physical growth and cognitive outcomes at 3-years

The SES and relationship domains had the greatest effect on cognition at 3-years ( Figure 2C, Figure 3C–D and Extended Data-Table 2 ^40^); each additional adversity experienced within these domains was associated with a decrease of 0·19 (95% CI: -0·24, -0·14; p < 0·001) and 0·18 (95% CI: -0·25, -0·10; p < 0·001) units in DEEP z-scores *.* Maternal stress led to a moderate decrease of 0·09 units per unit stressor (95% CI: -0·16, -0·03; p = 0·007), whilst the child domain had no effect (decrease for every additional child factor = 0·01 units (95% CI: -0·07, 0·05; p = 0·767).

All adversity domains, including child factors, showed significant inverse associations with HAZ and WAZ ( Figure 3C–D and Extended Data-Table 2 ^40^). The effects of SES, Maternal stress and Relationships on cognition and physical growth outcomes were comparable (Extended Data-Table 2 ^40^).

### Prospective association between hair cortisol levels and growth and cognition

**Figure 4.**
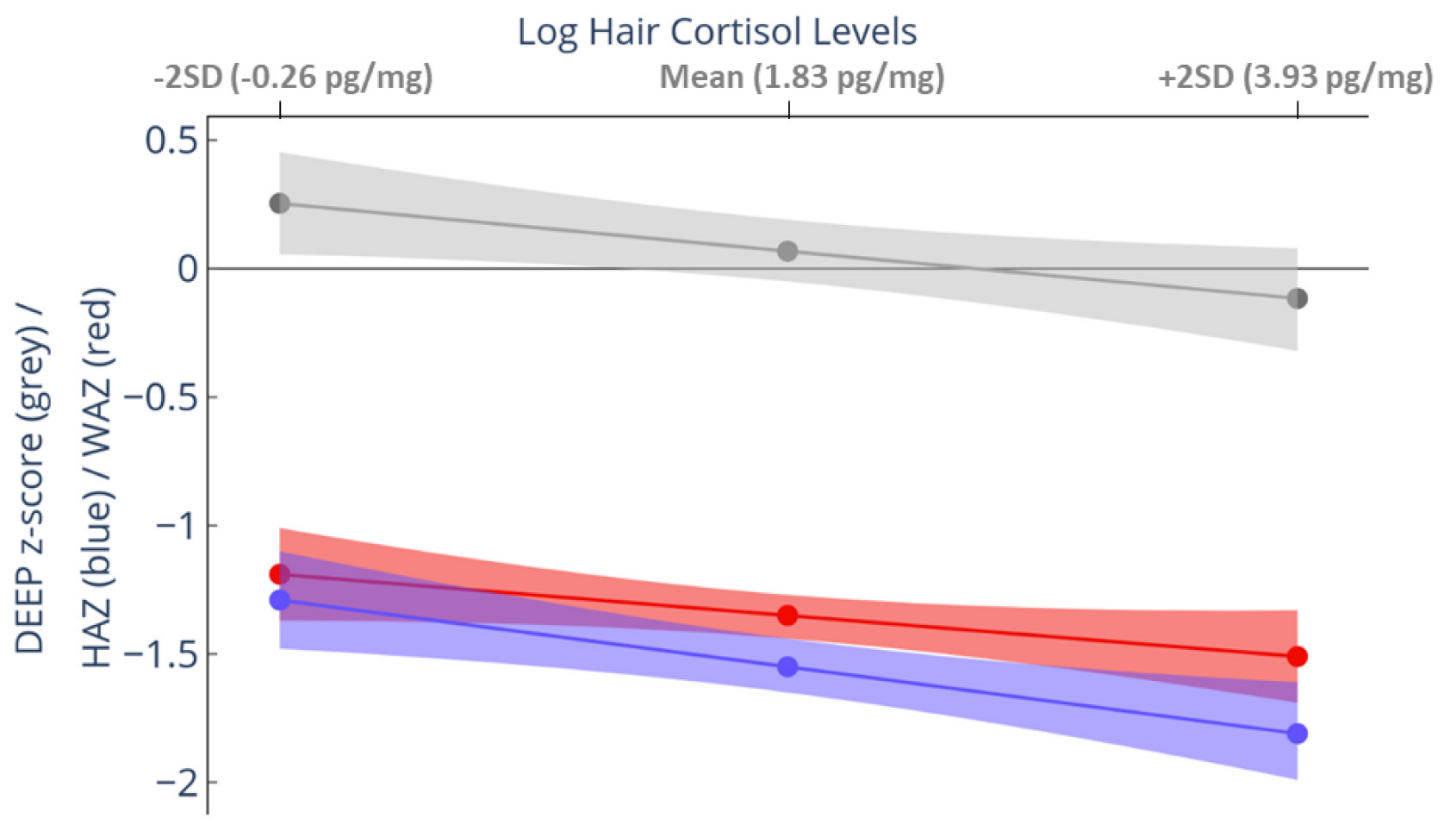
Relationship between hair cortisol concentration measured at 12 months and growth and development measured at 3 years. The predicted mean (95% confidence interval [CI]) outcome scores were computed from mixed-effects linear regression models adjusted for gender and age at 36-month assessment. The mean predicted height-for-age z-score (HAZ) (blue), weight-for-age z-score (WAZ) (red), and Developmental Assessment on an E-Platform (DEEP) z-score (grey) with 95% CI (shaded area) is plotted against the mean and ± 2SD of log hair cortisol levels in the sample.

Four outliers for hair cortisol concentration were winsorized to 3 SDs above the mean. Hair cortisol concentration at 12-months showed a significant inverse association with cognition at 3-years. For every unit increase in log hair cortisol levels, we observed a decrease of 0·09 (95% CI: -0·16, -0·01; p = 0·04) units in DEEP z-score ( Figure 4 and Extended Data-Table 3 ^40^). The predicted mean DEEP z-score of children in the top 5% of hair cortisol levels was -0·203, whereas for those with the least hair cortisol levels (minimum detectable limit) was +0·363, resulting in a difference of 0·56 SDs between the extreme groups. A similar relationship was observed between hair cortisol levels at 12-months and both HAZ and WAZ. For HAZ, there was a decrease of 0·12 units for every unit increase in log hair cortisol levels (95% CI: -0·20, -0·04; p = 0·005). However, the decrease for WAZ was not significant (p = 0.142; Extended Data-Table 3 ^40^).

### Exploratory mediation analysis

**Figure 5.**
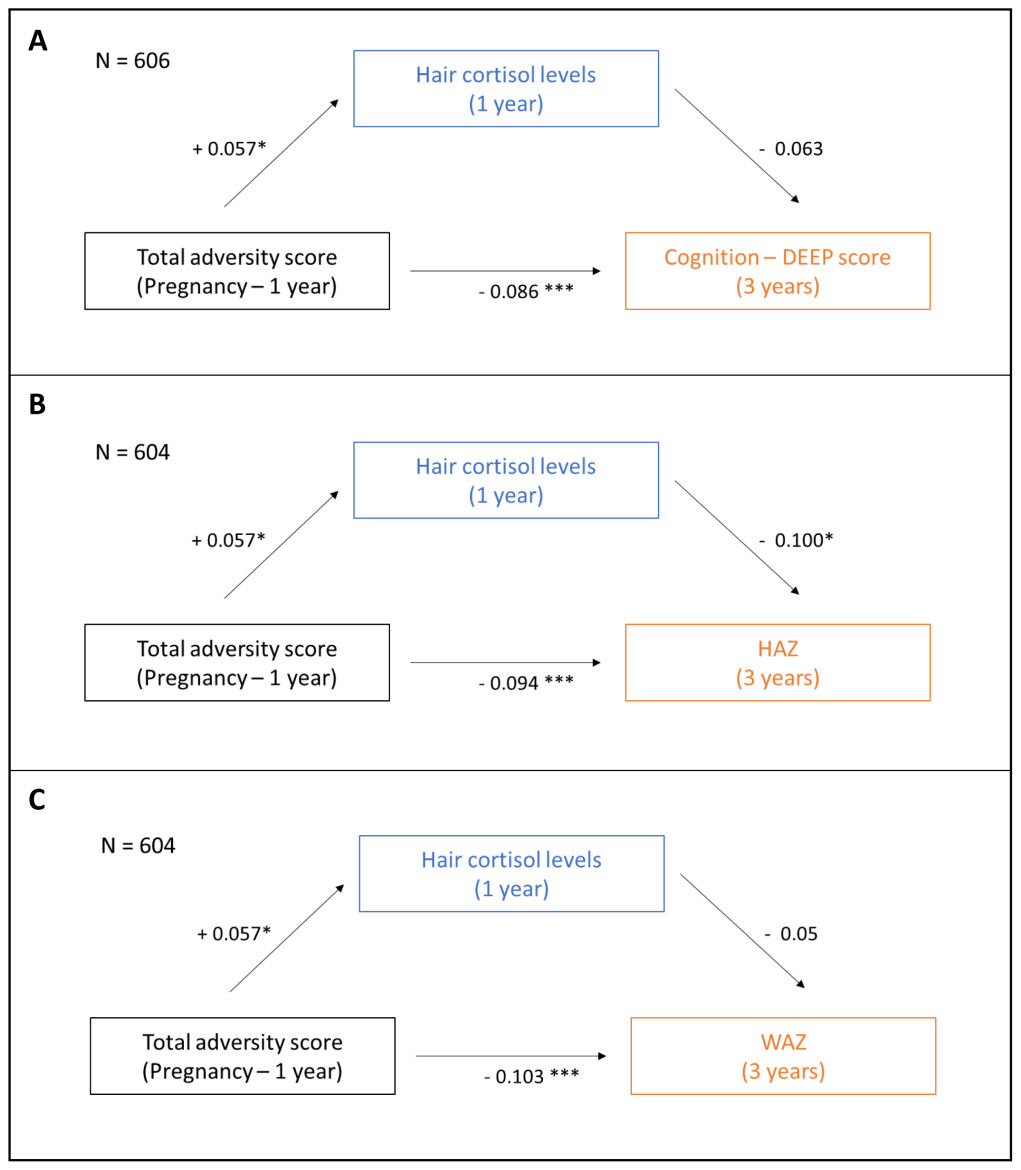
Exploratory mediation analysis to assess the putative mediating role of hair cortisol concentration on the association between cumulative adversity total score and child outcomes. The putative mediating role of hair cortisol concentration measured at 12 months on the association between early adversities measured during pregnancy and through the first year of life on ( **A**) cognition, ( **B**) height-for-age-z-score (HAZ), and ( **C**) weight-for-age-z-score (WAZ) measured at 3 years was assessed using the ‘mediation’ package in the R statistical software. Effect estimates for total and indirect effects are indicated. *p < 0.05, ***p < 0.0001.

Table 3 and Figure 5 report the results from the analysis exploring the putative mediating role of hair cortisol concentration on the associations between cumulative adversity and physical growth and cognition. Before running the ‘mediation’ package in R, missing values were omitted from the full dataset to generate an analytic sample of N = 606 and 604, respectively, for the cognition and growth outcomes respectively (see Figure 5). We found no evidence of statistically significant mediation by hair cortisol concentration on the association between cumulative adversities and cognition or WAZ (ACME = -0·004, p = 0·086 for cognition and ACME = -0·003, p = 0·16 for WAZ). On the other hand, hair cortisol concentration was found to partially mediate the association between cumulative adversity total score and HAZ (ACME = -0·006, p = 0·014 and ADE = 0·089, p < 0·001); the proportion of effect that was mediated was small (0·06, p = 0·014).

**Table 3.** Exploratory mediation analysis to assess the mediating role of hair cortisol concentration on the association between cumulative adversity and child outcomes. This table reports the estimates for the Average Causal Mediation Effect (ACME), Average Direct Effect (ADE), the Total effect and the Proportion of effect mediated (Ratio of ACME and ADE) for the association between cumulative adversity and three child outcomes – cognition, height-for-age-z-score (HAZ) and weight-for-age-z-score (WAZ) – see Figure 5. p-values for each estimate are indicated within parenthesis. Estimates with p < 0.05 are highlighted in bold.

	DEEP (p-value)	HAZ (p-value)	WAZ (p-value)
ACME	-0·004 (0·086)	**-0·006 (0.014)**	-0·003 (0·16)
ADE	**-0·082 (< 0·001)**	**-0·089 (< 0·001)**	**-0·102 (< 0·001)**
Total effect	**-0·086 (< 0·001)**	**-0·094 (< 0·001)**	**-0·104 (< 0·001)**
Proportion mediated	0·04 (0·086)	**0·06 (0.014)**	0·02 (0·16)

## Discussion

This study prospectively evaluated the associations between early adversities experienced during pregnancy and through the first year of life, and later physical growth and cognition outcomes measured when children turned 3 years of age. We observed large and significant differences in both physical growth and cognitive outcomes between groups experiencing none versus 8+ concurrent adversities. The difference in mean HAZ and WAZ scores between these extreme groups were approximately one standard deviation, and the difference in cognitive outcomes were 0·7 SDs. These relationships were dose-dependent with no ceiling effects, and echo the results seen in the same sample when outcomes were measured at 18 months ^36^, suggesting that the impact of early adversities are sustained over time. The heterogeneity in study methods and participant characteristics make it hard to compare these estimates with the literature ^47– 49^; however, our results highlight the persistent effects of early adversities on later physical growth and cognition measured up to three years later, thereby aligning with a large body of literature demonstrating the long-term and detrimental effects of early adversities and chronic stress on child outcomes ^22, 50^.

This is the first large-scale study to explore the putative mediating role of hair cortisol concentration on the associations between early adversities and physical growth and cognition outcomes in preschool children. We found that 12-month hair cortisol concentration was inversely associated with cognition and linear growth (HAZ) at 3-years, but not significantly with WAZ. However, while hair cortisol marginally mediated the association between early adversities and HAZ, no significant mediation effects were observed for WAZ or cognitive outcomes. Therefore, while these findings support dysregulation of the HPA axis as an important risk factor for poor child outcomes ^51^, they do not support our hypothesis that hair cortisol concentration is a key mediator underlying the established relationship between early adversities and child outcomes. Three recent studies also found no evidence of mediation by hair cortisol concentration on the association between early adversities and child mental health at 3-years, supporting our results ^52– 54^. However, given the long standing and well-accepted hypothesis that chronic stress resulting from early adversities leads to dysregulation of the HPA axis ^55^, ultimately resulting in poor cognitive outcomes, future research should explore the role of other potential mediators linked to the stress pathway ^56^, as well as other mechanisms implicated downstream of early adversities such the immune, metabolic and epigenetic pathways ^57^.

Among the different sub-types of adversities assessed, the SES and Relationship subscales were found to have the largest effects on cognition and growth at 3-years. This is unsurprising, since factors associated with low SES have consistently been demonstrated to be detrimental to child growth and development ^58^. Similarly, the effects of the Relationship subscale, which measured the quality of nurturing relationships and learning opportunities in the home, supports the growing body of work which demonstrate the buffering effects of secure and responsive relationships against the negative impacts of early adversities and vice-versa ^59– 61^.

Contrary to previous findings at 18-months of age, cumulative adversities in the Child domain were not associated with cognitive outcomes at 3-years, although it is noteworthy that this domain showed the weakest association at 18-months ^36^. Recent literature suggests that different types of adversities may have differential effects on child outcomes ^62– 64^. For example, while adversities related to deprivation (neglect or material deprivation) may lead to poor cognitive outcomes, threat (physical and emotional abuse) is more likely to cause atypical social-emotional development ^64^. The cumulative prevalence of threat inducing factors (e.g. bullying by an older child) was considerably higher (50.1%) in our sample compared to neglect (6.3%), possibly explaining why Child factors measured at 12 months were not related to cognitive outcomes at 3 years. Interestingly, however, they still had significant effect on HAZ and WAZ outcomes at 3 years.

One of the key strengths of this study was the use of a scalable tablet-based tool (DEEP) to assess cognition in 3-year-old children which made it possible to assess cognitive outcomes in this large sample. Other strengths include the use of a population based sample from an LMIC that is representative of the demographic profile of the region from where it was recruited, a prospective study design, the use of validated measures for exposure and outcome assessments, measurements of a large number of adversities that are contextually relevant to the population and life-stage studied, and the analysis of the effects of both total and domain specific cumulative adversities on growth and cognition in preschool children. Limitations include no further adversity or stress assessments after 12-months of age which may confound the impact of recently experienced adversities – we intend to do this in due course.

## Conclusions

Our results add to the growing body of literature that demonstrate the negative impact of early adversities on growth and cognition across infancy ^65^ and early childhood ^66– 69^. It also provides the first demonstration of a significant prospective association between hair cortisol concentration and linear growth (HAZ) and cognition in the first 3 years of life, as well its differential mediating role in the association between early adversities with HAZ versus cognitive outcomes. Continued follow-up of the SPRING cohort into adolescence and adulthood will provide a platform to describe the long-term consequences of early adversities and stress on physical and mental health across the life course in a LMIC context. Our results make it imperative that we continue to estimate the prevalence and impact of early adversities on concurrent and future health outcomes so as to mitigate their adverse effects through contextually appropriate interventions. The Sustainable Development Goals (SDG) ^70^ and the Nurturing Care Framework ^71^ provide excellent frameworks to design and operationalize this effort.

## Data availability

### Underlying data

LSHTM Data Compass: Dataset for: The effect of cumulative early life adversities, and their differential mediation through hair cortisol levels, on childhood growth and cognition: Three-year follow-up of a birth cohort in rural India. https://doi.org/10.17037/DATA.00002753 ^40^.

This project contains the following underlying data:

SPRING-REACH_dataset.csv

### Extended data

LSHTM Data Compass: Dataset for: The effect of cumulative early life adversities, and their differential mediation through hair cortisol levels, on childhood growth and cognition: Three-year follow-up of a birth cohort in rural India. https://doi.org/10.17037/DATA.00002753 ^40^.

This project contains the following extended data:

- 2373_UserGuide.html (user guide and codebook for dataset)
- Adversity_growth_development in .docx and .pdf formats (Extended data Tables 1–3, Figure 1)

Data are available under the terms of the Creative Commons Zero "No rights reserved" data waiver (CC0 1.0 Public domain dedication).
