# Supplementary material for: The effect of cumulative early life adversities, and their differential mediation through hair cortisol levels, on childhood growth and cognition: Three-year follow-up of a birth cohort in rural India
Source: Wellcome Open Res. 2022 Aug 26;7:74. Originally published 2022 Mar 3. [Version 2] doi: 10.12688/wellcomeopenres.17712.2 (PMC9096148; doi:10.12688/wellcomeopenres.17712.2)
Supplement: Peer review history

# Peer review: Reviewer response for version 2

**DOI:** 10.21956/wellcomeopenres.20273.r52178

**Published:** 2022-08-30

**Authors:** Helen Minnis

**Affiliations:** 1 Institute of Health and Wellbeing, University of Glasgow, Glasgow, UK

No further comments.

Is the work clearly and accurately presented and does it cite the current literature?

Yes

If applicable, is the statistical analysis and its interpretation appropriate?

Yes

Are all the source data underlying the results available to ensure full reproducibility?

Yes

Is the study design appropriate and is the work technically sound?

Yes

Are the conclusions drawn adequately supported by the results?

Partly

Are sufficient details of methods and analysis provided to allow replication by others?

Yes

Reviewer Expertise:

Child and adolescent psychiatry, adversity research epidemiology, randomised controlled trials.

I confirm that I have read this submission and believe that I have an appropriate level of expertise to confirm that it is of an acceptable scientific standard.

# Peer review: Reviewer response for version 1

**DOI:** 10.21956/wellcomeopenres.19599.r50126

**Published:** 2022-05-11

**Authors:** Helen Minnis

**Affiliations:** 1 Institute of Health and Wellbeing, University of Glasgow, Glasgow, UK

It was a pleasure to read this clearly written account of a wonderful study, in which adversities experienced by children through pregnancy and the first year of life were measured in more than 1300 children, living in rural India, at the age of twelve months. At age 3 years, more than 80% of the cohort were followed up and height-for-age, weight-for-age and cognition were measured alongside hair cortisol which was examined as a potential mediator between age 12-month cumulative adversities and age 3 year outcomes.

 As the authors point out, there has never previously been a large-scale study exploring the putative mediating role of hair cortisol on the associations between early adversities and physical growth and cognitive outcomes in preschool children.  Excellent prospective longitudinal studies of infant development of this scale are rare worldwide and this is a very welcome addition to the field.  This is a rare and precious sample because of the high proportion of children experiencing extremes of adversity alongside a wide variance in experiences of adversity across the sample.  Lack of variance in experiences of adversity (especially few participating children with high cumulative adversity) has been a real problem for prospective longitudinal cohorts attempting to understanding associations between ACEs and outcomes during childhood in White, Educated, Industrialised, Rich and Democratic (WEIRD) countries.  The only downside of this cohort is the lack of very clear information about abuse and neglect other than by siblings. This is not a criticism since this is almost certainly inevitable if accurate child protection recording/records are lacking in this geographical area – but is perhaps worth mentioning as a limitation.  On the other hand, I was very impressed by the pragmatic way in which adversities were measured and the child centred way in which direct adversities had been considered.

- The introduction provided a comprehensive and clear justification for the study.
- The methods were also clear. A little bit more information about how the SPRING-ELS sub-study was targeted and recruited might have been helpful although this is not a major issue as this is described in previous publications. I found Table 1 a little difficult to understand in terms of how points a to e related to the four domains – some clarification would be helpful.
- The team are to be congratulated on a fantastic retention rate and extremely few withdrawals.  There was also a clear demonstration that there were no major systematic bias due to attrition. 
- The results were, on the whole, very clear. Perhaps more was made of Figure 2B than was necessary – did it add anything to the data displayed in Figure A?
- The discussion was very clearly written.  In my view, the only thing missing was a consideration of the potential for reverse causality.  Although cognition at 12 months of age would have been hard to measure reliably (so reasonable not to have done this), 12 month cognition was likely highly correlated with cognition at 3 since cognition is generally a highly stable trait in the absence of extreme adversity.  Cognitive impairment can be stressful for both children themselves and for their families and can lead to adversity through higher rates of abuse, neglect and bullying – and through families having to look after children closely for longer, with potential impairments to income and social integration. Worth mentioning this as something to perhaps consider in future research.

Is the work clearly and accurately presented and does it cite the current literature?

Yes

If applicable, is the statistical analysis and its interpretation appropriate?

Yes

Are all the source data underlying the results available to ensure full reproducibility?

Yes

Is the study design appropriate and is the work technically sound?

Yes

Are the conclusions drawn adequately supported by the results?

Partly

Are sufficient details of methods and analysis provided to allow replication by others?

Yes

Reviewer Expertise:

Child and adolescent psychiatry, adversity research epidemiology, randomised controlled trials.

I confirm that I have read this submission and believe that I have an appropriate level of expertise to confirm that it is of an acceptable scientific standard.

# Response

**Published:** 2022-08-18

**Authors:** Debarati Mukherjee

**Affiliations:** Indian Institute of Public Health, Bengaluru, Public Health Foundation of India, India

Thank you for your kind review of this article. Please find our responses below:

- *The methods were also clear. A little bit more information about how the SPRING-ELS sub-study was targeted and recruited might have been helpful although this is not a major issue as this is described in previous publications. **I found Table 1 a little difficult to understand in terms of how points a to e related to the four domains – some clarification would be helpful.***

Apologies for the confusion. Points a to i are footnotes for each of the adversity measures listed in the table, that were then subsequently categorized into the four domains.

 For example the footnote for Point **a** is *“Socio-economic status (SES) score calculated with principle components analysis using household demographics and animal & asset ownership”* - which is an explanation for how we identified the subset of participants who met the criteria of experiencing this adversity: *“* Socioeconomic status: lowest quintile of asset index (*E) *^a^* *^”^*. This adversity was categorized into the SES domain as shown in the table.

 Similarly the footnote for Point **e** is “If woman reported husband drinking alcohol, answered yes to question: “does this cause any problems for you”. This was the criteria used to identify participants to be included in the adversity factor defined as “Husband’s alcohol use causes problems for mother ^e^^”^. This adversity was categorized into the Maternal Stress domain as shown in the table.

- *The results were, on the whole, very clear. Perhaps more was made of Figure 2B than was necessary – did it add anything to the data displayed in Figure A?*

Although the overall trend of the results were the same, Fig. 2B accounts for the fact that adversities are known to cluster within households, and therefore it may be likely that the different adversity measures included in our total adversity score are highly correlated (always occurring together). This may introduce a bias through ‘double counting’ co-occurring adversities. To overcome this potential bias, we used a principle component analysis (PCA) approach to determine the linear combination of factors that contributed to the maximum variance in the data, to avoid misinterpretation due to underlying collinearity between adversity measures. The similarity in results presented in Figs 2A and 2B demonstrate the robustness of the association between early adversities and poor child outcomes, irrespective of the method of analysis.

- *The discussion was very clearly written.  In my view, the only thing missing was a consideration of the potential for reverse causality.  Although cognition at 12 months of age would have been hard to measure reliably (so reasonable not to have done this), 12 month cognition was likely highly correlated with cognition at 3 since cognition is generally a highly stable trait in the absence of extreme adversity.  Cognitive impairment can be stressful for both children themselves and for their families and can lead to adversity through higher rates of abuse, neglect and bullying – and through families having to look after children closely for longer, with potential impairments to income and social integration. Worth mentioning this as something to perhaps consider in future research.*

While we completely agree with your point. However, there were very very few children who were reported to have a developmental delay or disorder in our sample.

# Peer review: Reviewer response for version 1

**DOI:** 10.21956/wellcomeopenres.19599.r49048

**Published:** 2022-03-28

**Authors:** Joe Piper

**Affiliations:** 1 Centre for Genomics and Child Health, Blizard Institute, Queen Mary University of London, London, UK

This is an elegant and interesting study examining how early adversities in the SPRING RCT in rural India measured at 12 months impact growth and cognition at 3 years. It also explored the association and mediation of hair cortisol concentration. Cumulative adversities in all domains (except the child domain) were inversely associated with growth and cognitive measures. An association of increasing 12 month hair cortisol concentration was observed with decreasing cognition and HAZ scores, but not WAZ. Mediation analysis suggested that hair cortisol concentration partially mediated the association between cumulative adversities and HAZ, but not for cognition. Hence this studies concludes that other biomarkers, and alternative pathways should also be explored in the future. I would recommend publication with a few minor corrections as detailed below:

- The second sentence in the introduction is too long and needs dividing: *Early adversities could have life-long consequences such as mental ill-health ^2, 3^, poor school readiness and academic achievement ^4^, and possible adoption of maladaptive behaviors **^5^. These **not only compromise growth, development and health through childhood.*
- The proportion of imputation of missing values is generally small with the exception of relationship domain: this could be pointed out in the discussion.
- 606 out of 845 hair cortisol measurements were used: have the authors considered a sensitivity analysis using all the hair cortisol measurements with the missing data imputed? It would be good to know if a sensitivity analysis on this was performed, or if not, why this was not feasible.
- For the winsorized hair cortisol measurements that were defined as 3 SD’s above the mean, was there any other explanation for these high cortisol measurements? Were they representative of particularly high levels of adversity, or attributed to measurement error? An extra line explaining this would be helpful.
- A brief description of the mediation analysis techniques used in the R mediation would also improve the paper.
- Figure 4’s caption should be expanded slightly to improve its clarity. This could include another line describing how increasing hair cortisol was associated with worse growth and DEEP-Z score.

Is the work clearly and accurately presented and does it cite the current literature?

Yes

If applicable, is the statistical analysis and its interpretation appropriate?

Yes

Are all the source data underlying the results available to ensure full reproducibility?

Yes

Is the study design appropriate and is the work technically sound?

Yes

Are the conclusions drawn adequately supported by the results?

Yes

Are sufficient details of methods and analysis provided to allow replication by others?

Yes

Reviewer Expertise:

nutrition, paediatrics

I confirm that I have read this submission and believe that I have an appropriate level of expertise to confirm that it is of an acceptable scientific standard.

# Response

**Published:** 2022-08-18

**Authors:** Debarati Mukherjee

**Affiliations:** Indian Institute of Public Health, Bengaluru, Public Health Foundation of India, India

Thank you for your kind review of this article. Please find our responses below:

- *The second sentence in the introduction is too long and needs dividing: Early adversities could have life-long consequences such as mental ill-health ^2^ ^,^ ^3^ , poor school readiness and academic achievement ^4^ , and possible adoption of maladaptive behaviors **^5^** **. These **not only compromise growth, development and health through childhood.*

Thank you for this suggestion. This has been edited in the new version of the manuscript.

  

- *The proportion of imputation of missing values is generally small with the exception of relationship domain: this could be pointed out in the discussion.*

The following sentence was included in the section describing the strengths and limitations of the study within the discussion:

 Although a few data points related to adversity measures were imputed, the proportion was very small (~2%) with the exception of the Relationship domain.

  

- *For the winsorized hair cortisol measurements that were defined as 3 SD’s above the mean, was there any other explanation for these high cortisol measurements? Were they representative of particularly high levels of adversity, or attributed to measurement error? An extra line explaining this would be helpful.*

We checked and found no association of children with very high cortisol levels (> 3SD) with their adversity scores. No clear reason for these high hair cortisol values were noted. The high values could have potentially resulted from measurement error, but we are unable to verify it.

  

- *A brief description of the mediation analysis techniques used in the R mediation would also improve the paper.*

A description of the mediation analysis has been provided in the methods section as copied below.

 Multilevel models, with cluster as the random effect, intervention allocation arm as the fixed effect and age at 3-year assessment and gender as confounders were generated between [1] exposure and outcome, [2] exposure and mediator, and [3] exposure and outcome adjusted for the mediator. Effect estimates were compared between models [1] and [3]. Causal mediation was determined using the ‘mediate’ function within the mediate package on models [2] and [3]. Results were tabulated to report the average causal mediated effect (ACME), the average direct (ADE), the total effect, and the proportion of effect that is mediated, along with their respective p-values.

  

- *Figure 4’s caption should be expanded slightly to improve its clarity. This could include another line describing how increasing hair cortisol was associated with worse growth and DEEP-Z score.*

The legend for Fig. 4 has been updated as suggested:

**Fig. 4: Relationship between hair cortisol concentration measured at 12 months and growth and development measured at 3 years.**

 The predicted mean (95% CI) outcome scores were computed from mixed-effects linear regression models adjusted for gender and age at 36-month assessment. The mean predicted HAZ (blue), WAZ (red), and DEEP z-score (grey) with 95% CI (shaded area) is plotted against the mean and ± 2SD of log hair cortisol levels in the sample. Increasing hair cortisol concentration was associated with worse growth (both HAZ and WAZ) and DEEP z-score (measure of cognitive abilities).
